# Alpha-synuclein oligomers: a new hope

**DOI:** 10.1007/s00401-017-1755-1

**Published:** 2017-08-12

**Authors:** Nora Bengoa-Vergniory, Rosalind F. Roberts, Richard Wade-Martins, Javier Alegre-Abarrategui

**Affiliations:** 1 Department of Physiology, Anatomy and Genetics, Oxford Parkinson’s Disease Centre, University of Oxford, South Parks Road, Oxford, OX1 3QT UK; 2 Montreal Neurological Institute, McGill University, 3801 Rue University, Montreal, QC H3A 2B4 Canada

**Keywords:** Alpha-synuclein, Parkinson’s, Oligomers, Aggregation, Detection-method

## Abstract

Alpha-synuclein is a protein implicated in Parkinson’s disease and thought to be one of the main pathological drivers in the disease, although it remains unclear how this protein elicits its neurotoxic effects. Recent findings indicate that the assembly of toxic oligomeric species of alpha-synuclein may be one of the key processes for the pathology and spread of the disease. The absence of a sensitive in situ detection method has hindered the study of these oligomeric species and the role they play in the human brain until recently. In this review, we assess the evidence for the toxicity and prion-like activity of oligomeric forms of alpha-synuclein and discuss the advances in our understanding of the role of alpha-synuclein in Parkinson’s disease that may be brought about by the specific and sensitive detection of distinct oligomeric species in post-mortem patient brain. Finally, we discuss current approaches being taken to therapeutically target alpha-synuclein oligomers and their implications.

## A brief history of alpha-synuclein and Parkinson’s disease

Over the past two decades, the pre-synaptic protein alpha-synuclein (a-syn) has been irrefutably tied to the neurodegenerative disorder Parkinson’s disease. First, a genetic link was found to associate a-syn to Parkinson’ disease; the point mutation in *SNCA* (A53T) was demonstrated to cause autosomal dominant Parkinson’s disease [126] and several other point mutations (A30P, E46K, H50Q, G51D and A53E) have since been shown to cause familial forms of Parkinson’s disease and dementia with Lewy bodies (DLB) [4, 79, 84, 119, 129, 167]. Later, duplication or triplication of the a-syn locus was described in several Parkinson’s disease and DLB families [18, 62, 70, 141] and in a handful of sporadic cases [2]. A gene dosage effect of the synuclein gene (*SNCA*) has been suggested due to the observation that patients with a triplication have an earlier onset, more severe and faster progressing disease compared to those harbouring duplication of the *SNCA* locus [18].

An equally important milestone in the field was the identification of a-syn as a component of Lewy bodies in patients with both familial and sporadic forms of the disease [6, 147], providing a direct link between different forms of the disease. Not only did this discovery draw attention to aggregated forms of a-syn as mediators of Parkinson’s disease pathogenesis, but also opened the door to the use of a-syn detection techniques for diagnosis and staging. Although this breakthrough occurred 20 years ago, research to date has not been able to describe the exact mechanism by which a-syn accumulates causes neuronal loss and leads to the development of the disease. Multiple lines of evidence now suggest that oligomeric species of a-syn, which are thought to precede the fibrillar aggregates found in Lewy bodies, are the culprits for neuronal degeneration in Parkinson’s disease [59, 162]. In this review, we will discuss the evidence supporting the toxicity of a-syn oligomers in Parkinson’s disease and possible mechanisms for this toxicity, current methods for neuropathological analysis of a-syn oligomer deposition, recent findings indicating the prion-like spread of a-syn and possible therapeutic avenues targeting a-syn oligomers.

## A-syn oligomers and neurotoxicity

### Lewy bodies: famously guilty or innocently accused?

The presence of fibrillar a-syn in Lewy bodies brought attention to the potential involvement of aggregated a-syn in Parkinson’s disease. However, several lines of evidence suggest that Lewy bodies themselves may be ‘innocent bystanders’ in Parkinson’s disease pathogenesis and that the toxic species of a-syn is in fact oligomers, which are precursors to Lewy bodies and not as readily detectable in histological sections [17]. First, Lewy bodies have been found post-mortem in neurologically normal individuals at the surprisingly high rate of approximately 10% in those over 60 [48, 118]. Second, the Lewy body load in patients has been shown to correlate poorly with the severity of symptoms such as cognitive impairment and dementia [26, 117]. Finally, Parkinson’s disease patients carrying familial mutations in the *parkin* gene, and some of those with the LRRK2 G2019S mutation, show neuronal degeneration in the absence of Lewy body formation [28, 50]. Together with direct evidence demonstrating the toxicity of oligomeric forms of a-syn, described in the following section, these observations suggest that Lewy bodies form as a protective mechanism by acting as a sink for toxic oligomers, sequestering them away from the cellular machinery [13, 108, 144].

### Structure and toxicity of a-syn oligomers

The term “oligomer” is widely used to describe aggregated a-syn that has not acquired a fibrillar conformation. The term itself is unspecific as to the molecular composition of a-syn oligomers, which encompass a wide spectrum of molecular weights, and vary in their beta-sheet content and exposed hydrophobicity. It was reported that while low molecular weight and unstable oligomers only had marginal seeding effects, large and stable oligomers were elongated and had high seeding properties [122]. Ghosh et al. have proposed that a-syn goes through a helix-rich intermediate before forming fibrils, which suggests oligomers could have a highly helical structure [52], and according to a recent biophysical study, a common feature a-syn oligomers is a “hollow cylinder” morphology [21]. Also, different mutations in the SNCA gene can influence the structure of their oligomers; A30P mutants form annular pore-like structures while A53T forms annular and tubular structures [80]. Oligomers developed through different protocols have also been shown to elicit different toxic effects: in agreement with Pieri et al., Danzer et al. described in 2007 that small annular oligomers were able to provoke calcium influx, caspase activation and cell death in vitro but had a low seeding capacity, whereas larger oligomers were not toxic but could seed further inclusions [30]. This highlights the heterogeneity of oligomers prepared through different protocols. It is also important to note that while experimental preparations of oligomers are highly enriched for this species, monomers, oligomers and fibrils (and probably other intermediate forms) live in equilibrium and that they constantly aggregate and disaggregate [29], making it difficult to categorically assign experimental effects to one species.

Amounting evidence indicates that a-syn oligomers are toxic. Not only are they present at the degenerating regions in Parkinson’s disease patients [140, 151] and transgenic models [53, 64, 66, 121], but direct experimental evidence shows their toxic properties. In vitro formed a-syn oligomers ectopically applied to cell cultures or formed due to over-expression of a-syn induce cell death [20, 30, 109, 149], which has been recapitulated in vivo in several studies. First, expression of a-syn mutants rationally designed to reduce fibril propensity and increase oligomer formation was toxic to immortalised cell lines, primary cultures of rat neurons and dopaminergic neurons of *C. elegans* and *Drosophila* [71]. Similarly, Winner et al. showed that expression of E57K and E35K a-syn resulted in conformationally trapped oligomers that were unable to fibrilize and induced neurodegeneration of dopaminergic neurons in the substantia nigra pars compacta (SNc) of rats [162]. In contrast, a-syn 30–110 that forms fibrils at a fast rate, did not display toxicity, indicating that oligomers are indeed the toxic species leading to TH-neuron loss in vivo [162]. The study of Winner et al. raises interesting insights into the possible presence and the potential for increased toxicity of ‘off-pathway’ oligomers, which do not form fibrils, in vivo. Off-pathway species, which have also been reported to form in vitro [27, 109], may bypass the cellular protective mechanisms of Lewy bodies and thus result in more cellular damage. Finally, a-syn bimolecular fluorescence constructs, which have been shown to form oligomers [113], were also shown to induce nigral degeneration upon injection of adeno-associated virus (AAV) particles containing the constructs into the rat SNc [35]. These studies suggest the formation of oligomers represent a toxic gain of function for a-syn.

It is important to note that although physiological a-syn has largely been considered a natively unfolded monomer that acquires alpha-helical secondary structure when interacting with phospholipids [157], it was recently suggested that native a-syn exists physiologically as a folded helical tetramer that is resistant to fibrillization and thus distinct from pathological oligomers [8, 34, 96, 156]. This hypothesis has been challenged using several techniques including in-cell NMR showing that a-syn behaves as a disordered monomer under native in vivo conditions [10, 14, 44, 150]. Interestingly, recent data from Burre et al. using FRET and cross-linking demonstrated that an equilibrium exists between monomeric cytoplasmic a-syn and multimeric membrane-bound a-syn that acts as a SNARE chaperone [14]. The conformation of physiological a-syn remains a contentious issue but understanding the identity of the native form, or whether there are multiple native conformers in equilibrium in the cell, is important to inform the development of potential anti-aggregation therapies for PD. Further research is essential to fully understand the native conformation(s) of physiological a-syn, the events that lead to its aggregation and the identity of the pathological aggregated species of a-syn.

## Mechanisms for oligomeric a-syn neurotoxicity

The mechanism of a-syn oligomer-induced neurodegeneration may involve the disruption of a variety of cellular processes implicated in Parkinson’s disease. We will briefly highlight only the most important processes since this has been reviewed elsewhere [63].

### Mitochondrial defects

The high energy demands and increased oxidative stress of dopaminergic neurons render them particularly sensitive to mitochondrial dysfunction [133]. In 2014 Plotegher et al. showed that mitochondrial morphology is disrupted by a-syn oligomers, which cause fragmentation of these organelles in vitro in SH-SY5Y cells [124]. In addition, a-syn oligomers were also shown to associate with the mitochondrial membrane in an A53T overexpressing transgenic mouse model of Parkinson’s disease that showed complex I impairments [22]. Unlike monomers and fibrils, oligomers were able to elicit mitotoxicity in primary mitochondria; they decreased the retention time of exogenously added calcium, promoted calcium-induced mitochondrial swelling and depolarization, and accelerated cytochrome c release [97]. Interestingly, it was very recently reported that astrocytes take up a-syn oligomers and degrade it via the lysosomal pathway, but this pathway can become saturated leading to mitochondrial fragmentation [89]. All these results show that a-syn oligomers are implicated in mitochondrial dysfunction across different models.

### Endoplasmic reticulum stress

When the endoplasmic reticulum (ER) is stressed and unable to function due to damage or protein accumulation, the unfolded protein response is activated, which removes misfolded proteins from the ER for degradation by the ubiquitin proteasome system. Accumulation of oligomers has been demonstrated in a transgenic mouse overexpressing A53T a-syn and in Parkinson’s disease brain tissue, resulting in chronic ER stress and impaired ER protein quality control [25]; furthermore, treatment with salubrinal, which alleviates ER stress, reduced oligomeric accumulation in the ER. Colla et al. also showed that a-syn oligomers associate with ER-membrane fractions in this murine model which was also true for material isolated from PD human brain; moreover, the accumulation was more noticeable in the brainstem than in the cortex, which is highly interesting since this distribution follows the neuropathology of PD [24]. While oligomers were able to induce toxicity and the activation of protective ER stress response factor X-box binding protein 1 in SH-S5Y5, monomers and fibrils were not able to elicit such effects [16]. These findings highlight the importance of removing or clearing pathological oligomers from the cell in order to avoid ER stress.

### Proteasomal effects

The ubiquitin proteasome system is one of the main mechanisms for the cell to remove misfolded proteins. This can be inhibited by a-syn oligomers: oligomers were shown to inhibit proteasomal activity, which was blocked by addition of antibodies that neutralized the interaction [87]. In agreement with these findings, A53T a-syn oligomers caused impairment of the proteasomal activity of PC12 cells, and this was partially reversed by addition of congo red, which is thought to prevent oligomerization [40]. Interestingly, a report by Lewis et al. showed that oligomerization of a-syn is promoted by its truncation by the proteasome [85]. Finally, inhibition of histone deacetylase 6 (HDAC6), which was previously shown to be involved in the response to cytotoxic ubiquitinated aggregates, increased the oligomeric content in vitro, while overexpression of HDAC6 produced the opposite effect [36]. These studies highlight the need to further study the relationship between a-syn oligomers and the proteasome in order to exploit its therapeutic potential.

### Non-cell autonomous roles: glial and inflammatory response

Astrocytes and microglia maintain neuronal homeostasis and participate in the clearance of the pathological aggregates [115], but it has also been shown that they enhance neurotoxicity due to their pro-inflammatory effects when in contact with a-syn [82]. The specific effects of oligomers have not been extensively tested; a report by Hoffmann et al. showed that fibrillar a-syn induced a more pronounced inflammatory response in microglial cells [61]. However, in a different report, oligomers were shown to activate pro-inflammatory signals in microglial cells in vitro and in vivo, and this was prevented by addition of a MAP kinase inhibitor [161]. Kim et al. demonstrated that a-syn oligomers lead to microglial inflammatory responses via TLR2 activation [74]. Another report by Zhang and collaborators also highlighted glial activation and production of reactive oxygen species in response to oligomer-like preparations of aggregated a-syn [168]. Astrocytes are able to take up a-syn oligomers and degrade it via the lysosomal pathway [89], but when this process is saturated it is possible that these cells only contribute to worsen the progression of pathology, leading to neuronal death. Finally, microglia isolated from adult mice or elderly humans display deficits in phagocytosis of a-syn oligomers compared with microglia from young mice or humans and that they secrete more TNF-alpha in response to a-syn insult [11]. Altogether, these data show that further studying the role that glia play in the oligomeric progression of PD is highly important in order to harness their potential role in PD therapeutics.

### Membrane damage

Membrane integrity is essential for the correct function of any cell type; therefore, its alterations can lead to cellular distress. A-syn oligomers can stabilize membrane defects accelerating membrane damage [19] and can alter membrane properties such as input resistance reducing neuronal excitability [72]. Dysfunctional membranes can also have an important impact on calcium homeostasis; some types of oligomers can lead to a cytotoxic calcium influx presumably by building pore-like structures [30]. Interestingly, depleting the extracellular space of calcium reduced the oligomer-induced cell death in another study, further highlighting the importance of membrane health and calcium homeostasis [3]. It was recently shown by two different groups that targeting the interaction between oligomers and membranes could elicit beneficial effects in different models [164, 166]. These results show that it is crucial to keep a physiological membrane status and that the interaction between a-syn oligomers and membranes leads to detrimental effects that can be reversed with the appropriate therapy.

### Autophagic and lysosomal dysfunction

a-syn accumulation has been linked to autophagic and lysosomal dysfunction, which may in turn lead to a-syn aggregation and production of more detrimental oligomers. Lee et al. originally showed that a-syn oligomers are cleared through the lysosome and that lysosomal block induces aggregation and toxicity in vitro [81], which is in agreement with the observations of Danzer et al. who showed that inhibition of lysosomal and autophagosomal fusion with bafilomycin led to an increase in exosomal a-syn while treatment with rapamycin reduced it [31]. Autophagy activation was also shown to reduce monomeric and oligomeric a-syn in neuronal cell lines elsewhere [90]. It was later demonstrated that bafilomycin treatment, which produced an increase in exosomal a-syn, resulted in inflammation and cellular damage in mice overexpressing a-syn under the PDGFB promoter [125]. Accordingly, overexpression of transcription factor EB (TFEB) was shown to correct lysosomal defects induced by the viral overexpression of a-syn and to downregulate the accumulation of oligomers in vivo [32]. All the studies above highlight the important role autophagy and lysosomal degradation play in a-syn degradation, opening exciting new options for Parkinson’s disease therapy.

### Synaptic dysfunction

Neurons require healthy synapses in order to carry out their biological function, and under normal circumstances a-syn has been shown to participate in vesicle clustering in the pre-synaptic compartment [49]. Synaptic dysfunction is thought to occur at the early stages of Parkinson’s disease [138] and oligomeric species of a-syn have been implicated in several pathways that could result in such an outcome. First, a-syn oligomers bind synaptobrevin, a component of the SNARE complex required for synaptic vesicle fusion, and prevent the formation of the SNARE complex [23]. In vivo, the result would be decreased neurotransmitter release and the possible ensuing loss of connectivity may lead to neuronal dysfunction and death. The trafficking of synaptic vesicles may also be negatively impacted by a-syn oligomers, which have been shown to decrease axonal transport by decreasing microtubule stability and impairing the interaction between kinesin and microtubules [128], as well as inhibiting tubulin polymerisation [20]. Interestingly, a-syn has just been reported as a novel microtubule dynamase, able to promote the nucleation of tubulin or microtubule catastrophe, depending on the concentration of free tubulin [15]. Parkinson’s disease-linked mutations in a-syn abrograted this activity, and although the effect of a-syn oligomers was not investigated, aggregation of the protein likely interferes with the ability of the tubulin binding domain (~amino acid 43–63) to interact with tubulin. Finally, it was recently shown that a dopamine catabolite, which is highly reactive, can promote oligomerization of a-syn and that these oligomers can produce a dopamine leak in vitro [123]. These studies highlight the fact that while the toxicity of a-syn oligomers is likely to mostly be through a toxic gain of function and its interference in multiple cellular pathway, at the synapse it can be interpreted as an over-active physiological function.

As the Golgi apparatus plays a role in the production of vesicles for synaptic release, Golgi dysfunction can have detrimental effects on synaptic output. Golgi fragmentation has also been observed as a result of oligomers formed by over-expression of a-syn in COS-7 cells [55]. A-syn oligomers may, therefore, decrease the pool of synaptic vesicles available as well as inhibiting their fusion to the membrane. Pore-like oligomers could also rupture synaptic vesicles, again leading to decreased neurotransmitter release, as well as permeabilising cell membranes, which could result in Ca^2+^ influx and excitotoxicity as was reported by Danzer et al. [30].

**Fig. 1:**
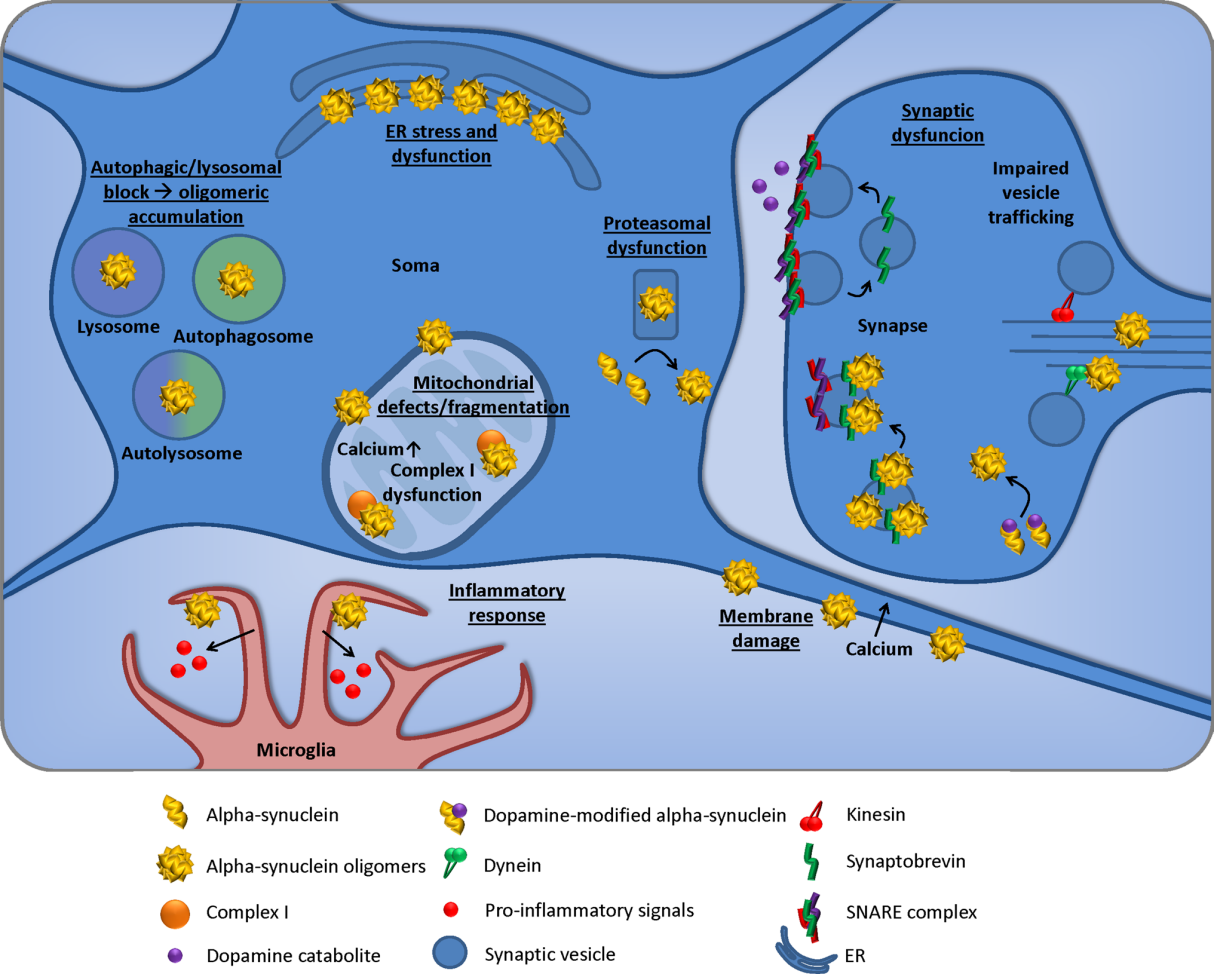
A-syn oligomers play a central role in the multi-factorial causes of Parkinson’s disease. A-syn oligomers, as well as other a-syn variants, actively participate in disrupting mitochondrial function, autophagy and lysosomal degradation, membrane homeostasis, ER function and synapses, and can induce inflammation. *Mitochondrial defects/fragmentation* have been observed after insult with a-syn oligomers. A-syn oligomers can also cause calcium accumulation and complex dysfunction inside the mitochondria. *ER stress and dysfunction* a-syn oligomers associate with ER membranes and cause ER stress. *Proteasomal dysfunction* while the proteasome normally degrades proteins, a-syn oligomers can cause proteasomal dysfunction, which in turn results in the accumulation of a-syn oligomers. *Inflammatory response* microglial cells respond to a-syn oligomers and produce neuro-inflammatory signals. *Membrane damage* oligomers can interact with the plasma membrane stabilizing previous damage and allowing the flow of calcium, which cause cellular distress. *Autophagic/lysosomal block* blocking the lysosomal and autophagic pathway results in oligomeric accumulation of a-syn and its detrimental effects. *Synaptic dysfunction* oligomers can bind to synaptobrevin, preventing SNARE complex formation and disrupting synaptic recycling of vesicles. They can also impair the interaction between kinsesin and microtubules, impairing vesicular transport, and finally, a dopaminergic catabolite has been shown to promote oligomerization of a-syn, leading to dopamine leak at the synapse

A-syn oligomers clearly negatively impact many cellular processes (Fig. 1), including membrane, proteasomal, mitochondrial and ER function, inflammation, autophagy and synaptic transmission, that are essential for the high metabolic and synaptic demands of dopaminergic neurons [154]. This suggests a central role in the multi-factorial causes of degeneration in Parkinson’s disease; future investigations are required to elucidate fully the unique or shared effects of structurally diverse oligomeric species on specific pathways, and techniques for the detection and differentiation of distinct a-syn oligomers will be key to this.

## A-syn seeds and spreads

Neuropathological examination of Parkinson’s disease progression suggested that a-syn pathology spreads through the nervous system following stereotyped patterns [12], which first led to the suggestion of a pathogenic agent ‘propagating’ through the nervous system. In 2008, it was shown that foetal dopaminergic neuronal grafts presented Lewy bodies 11–16 years after transplantation into Parkinson’s disease patients, suggesting a-syn pathology had spread from diseased neurons in the recipient’s brain to the grafted neurons [75, 86]. Consistent with these findings, intracerebral injection of brain extracts containing aggregated a-syn into a-syn-transgenic mice caused the development of lesions in anatomically linked regions of the brain, motor dysfunction and neurodegeneration [94, 107].

Recent work suggests that a-syn may be able to self-propagate between neurons in the brain, in a prion-like manner. The molecular events at each step in this process (the seeding of aggregates, the escape of aggregates from affected neurons and the entry of aggregates into naïve neurons) are only beginning to be elucidated. As in prion diseases, the ability of the molecule to self-interact and induce aggregation and conformational changes in another molecule is key, and it appears to be a feature of a-syn, demonstrated by the ability of a-syn aggregates to seed inclusions in vitro [58, 95] and in vivo [93, 103, 131]. A-syn oligomers display properties, including solubility and seeding ability, which make them likely candidates for mediating the spread of pathology. Small aggregate preparations produced by sonicating a-syn fibrils, known as pre-formed fibrils (PFFs), have been shown to propagate through non-transgenic mouse brain following injection into the striatum [93], while oligomers assembled from monomeric a-syn have also been described to have similar effects [131]. It is interesting to note that PFF treatment requires sonication, suggesting lower molecular weight species might also be implicated in their elicited effects.

In another study, injection of sarkosyl-insoluble a-syn isolated from the brains of dementia with Lewy bodies patients or fibrils produced in vitro mediated spread of a-syn pathology in the brain of non-transgenic mice [103]. This property is not unique to aggregates introduced directly into mouse brain: Helwig et al. induced a-syn over-expression in the medulla oblongata via viral transduction and demonstrated the presence of monomeric, oligomeric and fibrillar a-syn in medullary neurons, but crucially only monomeric or oligomeric species in pontine neurons, suggesting that non-fibrillar species of a-syn are preferentially transferred when expressed in the mouse brain [59]. In contrast to these other studies, 4-hydroxy-2-nonenal (HNE)-induced a-syn oligomers did not succeed in seeding inclusions in vivo [42], suggesting it is a specific conformationally distinct a-syn oligomer incapable of seeding pathology.

Further work has confirmed that different ‘strains’ of a-syn aggregates have varying propensities to propagate between neurons, in addition to their seeding ability. Peelaerts et al. generated a-syn oligomers, fibrils (which by electron microscopy more closely resemble PFFs, as opposed to full-length fibrils) and ribbons (fibrils with twists), which were all capable of crossing the blood–brain barrier and distributing themselves within the central nervous system after intravenous injection in mice [120]. While oligomers were the a-syn strain that diffused between brain regions more efficiently, fibrils and ribbons appeared to display higher toxicity [120]. The differences in prion-like activity and toxicity resulted in distinct pathologies and neurodegenerative phenotypes in the mice. While fibrils induced Lewy body-like inclusions, cell death and motor impairment, ribbons produced glial cytoplasmic inclusions, as found in MSA brain, in addition to Lewy body-like aggregates, and a phenotype resembling aspects of both MSA and Parkinson’s disease [120]. This inherent difference between a-syn strains in MSA and Parkinson’s disease were confirmed by Woerman et al. in cultured cells. Prion-like aggregates were isolated from MSA or Parkinson’s disease brain homogenates by phosphotungstate anion precipitation, a technique originally developed for studying infectious prion proteins [163]. While MSA brain-derived a-syn precipitates could seed a-syn aggregation in cultured cells, those from Parkinson’s disease brain could not, demonstrating that the a-syn species mediating the prion-like spread of pathology in Parkinson’s disease is distinct from MSA.

Interestingly, the spreading capacity of a-syn seems to be related to a-syn donor and acceptor species. While Luk et al. could not detect a-syn spread after mouse a-syn injections on a mouse a-syn KO mouse, Helwig et al. detected enhanced propagation of human a-syn, expressed from an AAV vector, on a mouse a-syn KO background. This suggests that (1) endogenous mouse a-syn is required for mouse a-syn spread, (2) human a-syn may have a higher propensity to spread than mouse a-syn, as it appears to lack the requirement for endogenous seeds in mice and (3) although human a-syn can form hybrid oligomers with mouse a-syn, these hybrid oligomers seem to form inefficiently and impede progress of spread. It is interesting to note that this is highly similar to a prion-like spread, where the interspecies barrier formed between infecting and host species is crucial [45]. This is confirmed by a recent paper by Luk et al. [92] where they showed that mouse and human a-syn cross-seeded with reduced efficiency, due to sequence differences.

Details are just beginning to emerge of specific molecular pathways that may mediate the secretion and uptake of a-syn seeds, although specific details on the implicated a-syn strain remain unexplored. Lee et al. identified a novel protein quality control mechanism (misfolding-associated protein secretion or MAPS), whereby under conditions of proteasome dysfunction, the deubiquitinase USP19 recruits misfolded proteins to the ER to be deubiquitinated and directed into late endosomes for secretion [83]. USP19 promoted the secretion of a-syn, suggesting that MAPS is an unconventional secretion pathway utilized by a-syn, particularly under conditions of proteasomal impairment, which has been repeatedly linked to Parkinson’s disease. It would be highly interesting to study which types of a-syn aggregates are being secreted (e.g. oligomers) and whether differential secretion exists for example among patient and control subjects. Once released by donor neurons, a-syn must be taken up by acceptor neurons in order to continue the cycle of spreading. Recently, lymphocyte-activation gene 3 (LAG3) was identified as a receptor for a-syn PFFs, allowing their entry into neurons and accelerating their spread throughout the mouse brain [100]. Although expression of LAG3 had not previously been reported in nervous tissue, knockout of LAG3 in neurons in vitro and in mouse brain clearly reduced a-syn propagation. An altogether different mechanism for a-syn spread was recently proposed by Abounit et al.: in their study they showed that a-syn is transmitted cell-to-cell via tunnelling nanotubules, which are transient nanostructures that allow cargo exchange between cells [1]. Lysosomal a-syn originally targeted for degradation was able to spread inside lysosomal vesicles from donor to acceptor cells, where it was then able to induce aggregation. It is tempting to speculate that owing to their smaller size and early aggregation properties, a-syn oligomers are likely candidates to be transported in this manner. These exciting new studies should inform novel drug targets in Parkinson’s disease, which if administered sufficiently early in the disease course, could prevent the devastating march of a-syn pathology through patients’ brains.

## Current methods for the detection of a-syn oligomeric pathology

**Fig. 2:**
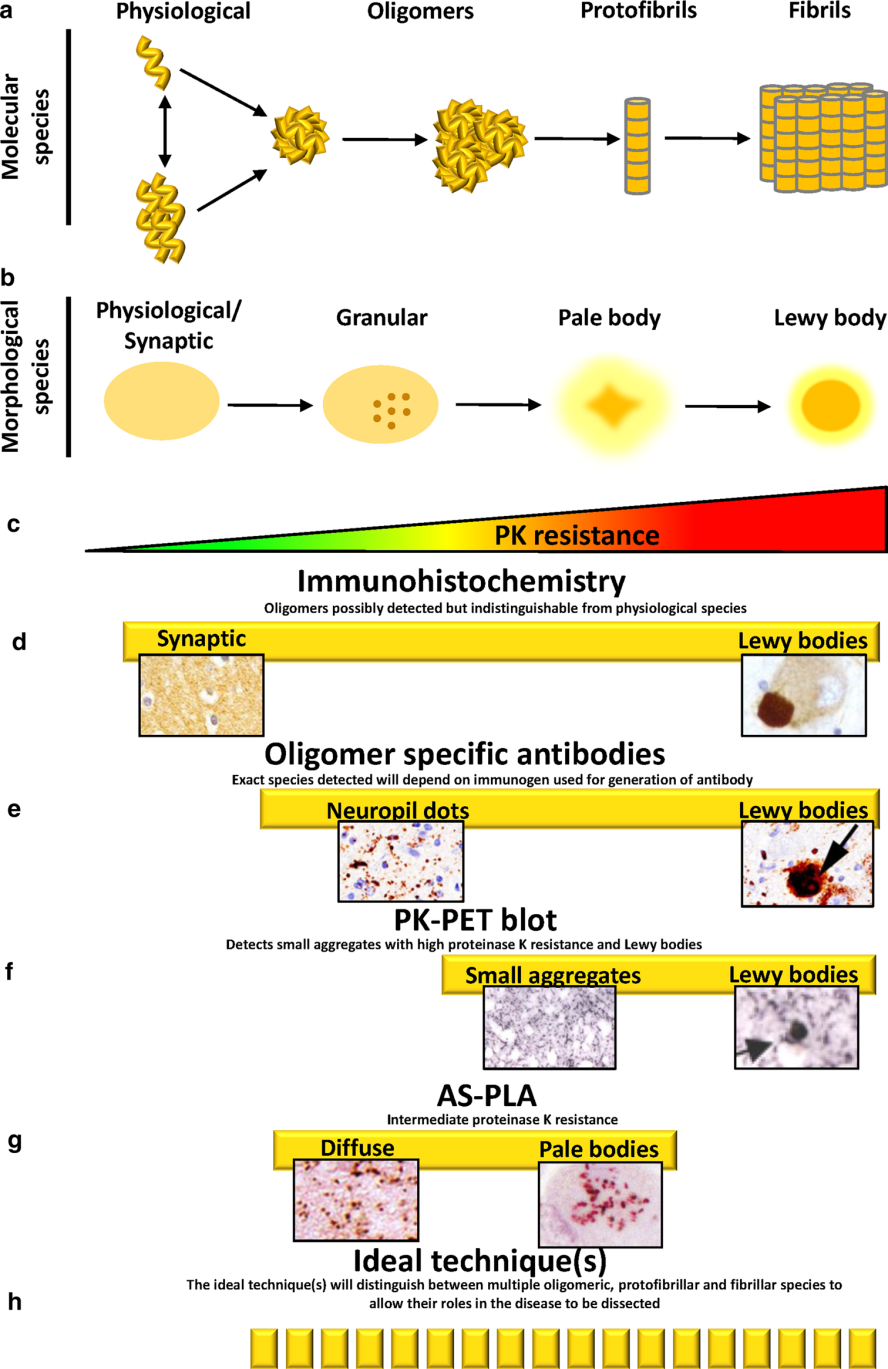
Methods for the histological detection of a-syn oligomers/aggregates. **a** Schematic of the pathway of a-syn aggregation at the molecular level, from physiological monomers/tetramers, through oligomers and protofibrils to fibrils. **b** Different morphological species of a-syn found in Parkinson’s disease brain, which have been described following AS-IHC. Physiological/synaptic staining of a-syn is present in the neuropil and indicates the localization of physiological a-syn at synapses. Granular staining is interpreted as the presence of small aggregates. Lewy bodies are the pathological hallmark of Parkinson’s disease and consist of highly aggregated a-syn, while pale bodies consist of less compacted a-syn and are thought to be a precursor to Lewy bodies. Note that the exact relationship between the morphological and molecular species of a-syn is not clearly elucidated. **c** The species represented in **a** and **b** display differential resistance to proteinase K (*PK*), with structures on the right showing the highest PK resistance. **d**–**h** Represent techniques currently in use for the in situ detection of a-syn aggregates in human brain. *Yellow bars* correspond to the type of species detected by each technique in relation to **a**–**c**. **d** a-syn immunohistochemistry is the classical method for the detection of pathological a-syn (i.e. Lewy bodies), but staining of physiological a-syn is difficult to interpret. While a-syn oligomers may be detected, it is impossible to distinguish them from physiological staining. **e** A variety of oligomer specific antibodies that detect Lewy bodies have been developed using in vitro generated a-syn oligomers as immunogens. Images adapted from Kovacs et al. [77], with permission from the publisher. **f** The PK-PET blot requires a harsh treatment with PK, leading to the detection of small aggregates with high PK resistance and Lewy bodies. Images adapted from Schulz-Schaeffer [138], with permission from the publisher. **g** AS-PLA detects oligomers with intermediate proteinase K resistance which distribute diffusely in specific neuroanatomical areas and accumulate in early lesions such as Pale bodies. **h** The ideal technique(s) will specifically detect and differentiate multiple a-syn species to allow the role of each in the disease to be dissected

In order to study the involvement of a-syn oligomers in the pathology of Parkinson’s disease, we require *bona fide* methods to detect oligomers in patient samples. While several methods are available to study a-syn oligomers in vitro and in cellular models (e.g. protein complementation assay), it is crucial to detect the species present endogenously in patients with Parkinson’s disease, both in brain samples and biological fluids. In this section we will discuss methods for the detection of a-syn oligomers in situ in tissue sections (summarised in Fig. 2) and in biological fluids.

### A-syn immunohistochemistry

The identification of a-syn as a major component of Lewy bodies, the histopathological hallmark of Parkinson’s disease, focused attention on aggregates of a-syn. Antibodies raised against purified Lewy bodies were shown to be specific against a-syn [6]. Furthermore, the use of a-syn immunohistochemistry (AS-IHC) allowed the visualisation of significantly more Lewy bodies with IHC compared to ubiquitin antibody detection, the previous standard for identifying Lewy bodies [146]. A-syn mediated detection of Lewy bodies is preferable to ubiquitin staining as Lewy bodies are not the only type of inclusion rich in ubiquitin [54]. AS-IHC is now the gold standard for the detection of Lewy bodies, and thus the pathological diagnosis of PD (Fig. 2d). However, as discussed earlier, Lewy bodies may form as a cellular protective mechanism to sequester damaging a-syn. Although suspected to be toxic, until now, a-syn oligomers have not been detected in situ, preventing interpretation of their role in Parkinson’s disease. The vast majority of studies that uncover pathogenic effects of a-syn oligomers have utilised in vitro formed oligomers, and so, the extent to which these oligomers recapitulate the structure and properties of those found in diseased brain remains unclear. In order to address these questions, the specific detection of a-syn oligomers in patient tissue is a pre-requisite.

### Conformation-specific antibodies

Numerous laboratories have used IHC with antibodies specifically raised against oligomeric forms of a-syn to elucidate the implication of a-syn oligomers in Parkinson’s disease. However, several of the antibodies produced to this end also detect oligomers composed of aggregated molecules of other amyloidogenic proteins, including A-beta, prion protein and lysozyme [73, 169]. The A11 antibody, for example, binds the beta-sheet edge in pre-fibrillar oligomers suggesting this is a common motif in pathogenic oligomers [73]. A shortcoming of the broad specificity of A11 is that it results in the detection of non-pathogenic proteins that contain the beta-sheet edge motif, such as IgG [165]. An additional problem of the detection of a-syn oligomers with conventional IHC is that oligomers formed in vitro have been used as the immunogen to raise oligomers-detecting antibodies, and therefore the variety of species detected by such antibodies may be not representative of the actual pathology. Furthermore, epitope changes following fixation are a particularly important consideration in this paradigm. The antibody mAb38F, which was raised by artificial immunization with recombinantly produced oligomers, was shown to recognise Lewy bodies in Parkinson’s disease and dementia with Lewy bodies (DLB) tissue, but no additional pathology was detected by this antibody in human tissue [41]. Furthermore, differentiating pathological a-syn staining from physiological staining is another caveat for this technique, which was further addressed with the development of the 5G4 antibody that was raised against amino acids 44–57 of a-syn [77] and was shown to bind high molecular weight beta-sheet rich a-syn oligomers [76]. Although the 5G4 antibody did not detect physiological a-syn and detected Lewy dots, Lewy bodies, Lewy neurites as well as granular structures in soma, the antibody did not detect pathology more efficiently than an antibody against full-length a-syn (4D6) [77] and did not identify previously unreported pathology by light microscopy [76] (Fig. 2e). This could be the result of a mixed specificity of this antibody due to cross-reactivity with fibrillar a-syn, which was reported in their 2014 study.

An antibody specific to a-syn phosphorylated at Ser129 demonstrated the presence of grain-like ‘Lewy dots’ in the neuropil around Lewy bodies [136]; this antibody highlighted the importance of a-syn phosphorylation in the pathology, but it unfortunately was still unspecific regarding the aggregation status of the detected species. Furthermore, in areas such as the cortex where a-syn is highly abundant at synaptic terminals, it is not possible to differentiate AS-IHC signal from pathological a-syn species from the physiological, synaptic staining of a-syn [138].

### Proteinase K-assisted detection of a-syn aggregates

To overcome the limitation of traditional AS-IHC in differentiating pathological and physiological forms of a-syn, tissue sections can be pre-treated with proteinase K (PK), which digests and eliminates non-pathological a-syn. Tanji et al. identified pre-synaptic aggregates in addition to extensive Lewy neurite and Lewy body pathology using this method [148]. However, such aggregates did not precede Lewy body formation and therefore are unlikely to represent early aggregated a-syn species associated with Parkinson’s disease. Using the PK paraffin embedded tissue blot (PK-PET blot) tissue is digested with PK for long periods (up to 8 h), and more extensive pathology is revealed compared to the approach used by Tanji et al. Heavy neuritic pathology, in addition to Lewy bodies, was revealed in the SNc of Parkinson’s disease patients using the PK-PET blot [111]. Small aggregates of a-syn shown to be localised to synapses were uncovered in the frontal and cingulate cortex of DLB patients and in the SNc of Parkinson’s disease patients with the PK-PET blot [78, 138] (Fig. 2f). Although earlier pathology is revealed with the PK-PET blot compared to traditional AS-IHC, the extremely proteinase K resistant species are likely to represent aggregates with high beta-sheet content that occur late in the misfolding process [106]. Furthermore, the extended digestion makes it highly variable and degrades the integrity of the tissue and therefore very poor neuroanatomical resolution remains for analysis.

### The a-syn proximity ligation assay

We recently described the development of the a-syn proximity ligation assay (AS-PLA) for the specific detection of a-syn oligomers [132]. The proximity ligation assay (PLA) was initially developed by Fredriksson et al. in order to sensitively detect protein levels [47] and were subsequently adapted for the detection of protein–protein interactions [56, 143]. PLA probes are generated from antibodies raised against the protein(s) of interest, one for each of the proteins involved in the putative interaction, which are conjugated to short oligonucleotides. If the probes bind interacting proteins, the oligonucleotides are sufficiently close to prime an amplification reaction, which can be detected by tagged oligonucleotides and observed as punctate signal, with each punctum representing an interaction. We adapted this technique in order to detect a-syn oligomers by using the same epitope-blocking anti a-syn antibody to generate both probes. AS-PLA selectively recognizes oligomers, while presenting minimal recognition of monomeric and fibrillar a-syn and none regarding other amyloid structures, for example A-beta oligomers, amyloid plaques and neurofibrillary tangles.

Using AS-PLA we were able to describe for the first time the presence of abundant diffusely deposited oligomeric pathology in the medulla and cingulate cortex of Parkinson’s disease post-mortem brain tissue. AS-PLA also labelled very early perikaryal aggregates in morphologically intact neurons that may precede the development of classical Parkinson’s disease, whereas brain stem LBs, considered heavily compacted late lesions, were only stained on their periphery, if at all, indicating that oligomers exist surrounding the more compact structures of LBs (Fig. 2g). Helwig et al. took advantage of this technique in order to dissect which species of a-syn preferentially transferred from cell to cell when expressed in the mouse brain [59]. In their study, AS-PLA enabled not only the detection of the spread of exogenous human oligomers, but also that of human-mouse oligomer hybrids, which highlights the need for therapies targeting the pathological oligomeric species. In summary, this technique, which has recently attracted a lot of attention [33, 60, 67, 160], has enabled for the first time to visualize oligomers in situ shining some light onto the role of a-syn endogenous oligomers in Parkinson’s disease.

### The ideal technique

The purpose of detecting alpha-synuclein oligomers directly in patient samples is to allow us to understand their association with disease. The ideal technique would allow the differentiation of many oligomeric species so that the properties of each and their contribution to disease can be studied, for example by comparing the abundance of particular species with clinical outcomes (Fig. 2h). This may be possible by generating antibodies against patient-derived oligomers and using a panel of such antibodies to identify pathological differences between patients. Another possible approach would be to modify the length of the oligonucleotide arms of AS-PLA probes in order to detect oligomers of variable sizes or to couple the antibodies to probes with different spacing sizes.

Altogether, with the tools described in this review (Fig. 2), we anticipate that the insights gained into early a-syn aggregation will open a window into the earliest stages of protein aggregation in Parkinson’s brain and will help to dissect the mechanisms by which a-syn oligomers mediate neurodegeneration in Parkinson’s disease.

## Detection of a-syn oligomers in biological fluids

The detection of a-syn oligomers in biological fluids (blood serum or plasma and cerebrospinal fluid) is of particular interest because of the potential for its use a biomarker of PD. Oligomeric detection may have uses as a diagnostic biomarker, as a biomarker of the progression of the disease, and in the future, perhaps as an index of response to novel therapies. The detection of a-syn oligomers in CSF and plasma has also proven a more reliable indicator of the presence of disease than the levels of total a-syn, which has led to contradictory findings [46]. Several groups have employed sandwich ELISAs to detect a-syn oligomers by using the same epitope-blocking antibody for both capture and detection. The use of a blocking antibody ensures that only one antibody molecule can bind to each molecule of a-syn so that no signal is generated from monomers. In all of these studies, higher levels of a-syn oligomers were detected in the plasma or CSF of Parkinson’s disease patients compared with controls [39, 116, 152]. This suggests that the presence of a-syn oligomers in biological fluids is a more reliable indicator of disease and measuring total levels of a-syn obscures the difference in oligomer levels because a variety of conformers are detected. Further refinements, for example increasing sensitivity, could be addressed by developing novel antibodies with higher avidities, or by adapting AS-PLA for use in biological fluids. PLA has previously been used to detect A-beta protofibrils in fluids, and it was shown to be 25-fold more sensitive than a sandwich ELISA targeting A-beta protofibrils [69].

ELISAs utilizing conformational antibodies targeting a-syn oligomers have also been developed. Majbour et al. [98] used an antibody that detected only aggregated (oligomeric and fibrillar) a-syn that they had previously generated [153]. In parallel, they undertook ELISAs targeting total a-syn or a-syn phosphorylated at serine 129. When applied to CSF samples from 46 patients with Parkinson’s and 48 healthy age-matched controls, the ELISA targeting aggregated a-syn demonstrated the highest sensitivity for detecting PD patients compared with those detecting total or phosphorylated alpha-synuclein (89 vs 74% and 54%, respectively), but the lowest specificity (52 vs 74% and 54%, respectively). Similarly, Williams et al. [159] applied two conformation-specific single chain variable domain antibody fragments (scFvs) that display sub-femtomolar sensitivity for a-syn oligomers to a phage-based capture ELISA they previously developed [158]. Elevated levels of a-syn oligomers were found in PD patients compared to controls or AD patients in brain homogenate, CSF and serum. However, the approaches described thus far show large variability and overlap in ELISA signal between controls and PD patients. Therefore, such assays at the current time may have limited capabilities as a biomarker readout.

The emerging hypothesis of the prion-like propagation of a-syn has led to the adaptation of techniques originally developed to detect pathogenic prion protein to detect oligomers of a-syn. The protein misfolding cyclic amplification (PMCA) assay was originally developed in 2001 in the laboratory of Claudio Soto [135] and has been shown to allow the detection of even a single pathogenic unit of prion protein [134]. The concept is based on the seeding capacity of prion proteins and incubates a suspected pathogenic sample with the physiological form of the protein. If pathogenic forms of the protein are present, seeding and aggregation of the monomeric protein will occur. Following the generation of new aggregates, a shaking step breaks them up to form new seeds, and the cycle is repeated many times to amplify the signal. Originally, the aggregates formed as a result of the presence of seeds were detected using western blotting [135], but this has now been adapted to allow for a more high-throughput format and thioflavin T fluorescence is used to assess the presence of aggregates. This assay design may preclude the detection of certain ‘off-pathway’ oligomers that do not aggregate further to from beta-sheet rich structures, but results thus far are promising.

Shahnawaz et al. [139] developed a PMCA for a-syn, first using in vitro formed a-syn oligomers and subsequently adapted for use in CSF. Levels of a-syn seeds in CSF from two cohorts of PD patients from Germany and Japan (76 patients) were compared to 65 controls (patients with other neurological diseases); they found that the assay was capable of detecting PD patients with a sensitivity of 88.5% and a specificity of 96.9%. Interestingly, of four controls in which the assay detected pathogenic a-syn, two were subsequently diagnosed with PD, suggesting the potential value of the assay for early diagnosis. Furthermore, the sensitivity and specificity of the assay for identifying PD patients is much higher than any previous reports of techniques to detect a-syn oligomers in biological fluids.

An adaptation of the PMCA, real-time quaking induced conversion (RT-QuIC) assay, has also been used to detect a-syn aggregates in CSF and proved to be even more sensitive [43]. Although the cohorts used were small (20 patients and 15 controls), the sensitivity was 95% and the sensitivity was 100%. Even more excitingly, three at-risk individuals who had confirmed REM sleep behaviour disorder all gave a positive signal in the assay, suggesting the assay may be used to detect the early stages of the disease.

Another exciting development is the finding that distinct biophysical properties (for example, proteinase K resistance) of a-syn seeds are retained after PMCA [65]. This may make it possible to build up an a-syn ‘seed profile’ of patient biological fluids and dissect whether there are different clinical outcomes associated with different a-syn species, as has recently been observed in Alzheimer’s disease patients [91].

The use of prion protein assays has provided the most sensitive and specific detection of pathogenic a-syn in biological fluids to date and provides hope that, following confirmation in large cohorts, a suitable biomarker may soon be able to be used in the clinic.

## The new hope: therapeutic potential

**Fig. 3:**
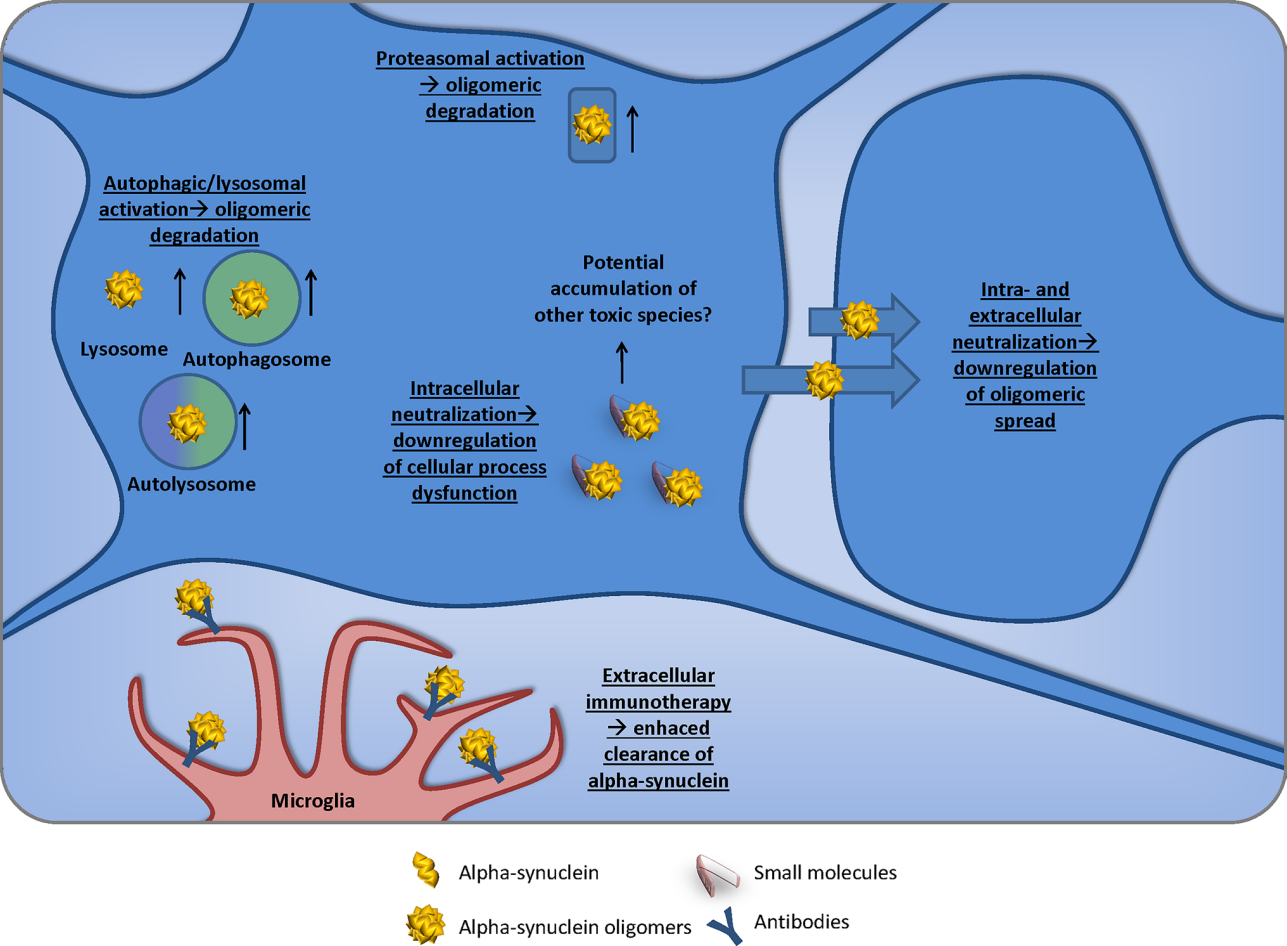
Therapeutic avenues leading to oligomer burden decrease. Small molecules and immunization have the potential to inhibit a-syn intra- and extracellular interactions and, therefore, prevent aggregation, degrade existing aggregated complexes and avoid detrimental interactions with cellular components. *Autophagic/lysosomal activation*: small molecules that upregulate autophagy and/or lysosomal degradation will result in the reduction of a-syn oligomers and its beneficial effects. *Proteasomal activation*: as autophagic and lysosomal activation, proteasomal activation will result in an increased degradation of a-syn oligomers. *Intracellular neutralization*: small molecules capable to bind and/or breakdown will result in important intracellular consequences. However, anti-aggregating small molecules have the potential side-effect of causing a build-up of intermediate but toxic species, so careful toxicological evaluation of these compounds is warranted. *Extracellular immunotherapy*: immunotherapy will recruit glial cells in order to induce phagocytosis and neutralization of toxic oligomers. Reducing the oligomeric pool via extra- and intracellular therapeutic pathways will provide a significant downregulation of the pathological burden and, therefore, reduce the spread of a-syn toxic species

The discovery that a-syn can transmit from cell to cell suggests that targeting a-syn oligomers is not simply limited to modifying the aggregation or clearance of a-syn, but also to its spreading process. This new paradigm has highlighted that the aggregation and accumulation of such species are key to their spread and, therefore, therapies affecting aggregation or clearance could have an important impact on oligomer burden (Fig. 3).

### Small molecules

Protein aggregation is a key feature in Parkinson’s disease and many other neurodegenerative diseases; therefore, many studies have focused on aggregation inhibitors. In vitro studies have shown that curcumin inhibited the formation of fibrils and disaggregated amyloid-beta and a-syn [112, 114, 155]. SH-SY5Y cells overexpressing the A53T mutant of a-syn and treated with curcumin showed a 33% reduction in aggregated A53T a-syn [114], and a toxicity assay in the same cellular model showed that curcumin was able to block pre-formed a-syn oligomer toxicity and destabilize pre-formed fibrils. However, the latter effect resulted in a significant increase in toxicity, suggesting the formation of a-syn toxic species after incubation of a-syn fibrils with curcumin [155]. This exemplifies one caveat of using aggregation inhibitors, which is that as we do not currently know the identity of the toxic species of a-syn it is difficult to predict the outcome of treatment with such compounds, which may inadvertently result in an increase in the toxic species. Fibrillar dissociation could, for example, lead to oligomeric a-syn release and its downstream detrimental effects.

Epigallocatechin-3-gallate (EGCG), a small molecule that been shown to inhibit the aggregation of several amyloidogenic proteins such as a-syn, amyloid-beta and huntingtin [9, 37, 38, 105] binds to unfolded native amyloid-beta and a-syn and promotes the formation of nontoxic oligomers that do not convert into amyloid fibrils [37]. Clinical trials using this molecule as an anti-aggregation agent have already started and are ongoing.

Molecular tweezers are molecules that resemble tweezers at the molecular structure level. The molecular tweezer CLR01 is able to bind lysine and inhibit the aggregation of a wide variety of proteins. In cell culture, CLR01 blocked effects of exogenous toxic oligomers of amyloid-beta40, amyloid-beta42, a-syn, islet amyIoid polypeptide, transthyretin, beta2-microglobulin and insulin [142]. In mice, CLR01 was able to cross the blood–brain barrier at 2% of blood levels and in triple transgenic Alzheimer’s disease mice CLR01 caused a reduction of amyloid-beta aggregates, hyperphosphorylated tau and microglia load [5]. Prabhudesai et al. showed that this molecule is able to inhibit the aggregation of a-syn in vitro and in a zebrafish model expressing human wild-type a-syn in neurons where CLR01 reduced apoptosis and improved embryo survival [127].

Indirectly targeting a-syn aggregation by modulating cellular pathways involved in the aggregation, such as molecular chaperoning, autophagy or the ubiquitin–proteasome system, is also an interesting therapeutic strategy. For example, the study of brain-permeable small molecules that can upregulate the levels or activity of chaperones such as HSP70 or HSP90, which are able to prevent a-syn oligomerization, could have a significant impact in oligomer burden [68, 104, 130].

### Immunization

Given the issues of unknown species resulting from the use of aggregation inhibitors or disaggregases, a therapeutic approach that can exploit the body’s endogenous systems to remove a-syn is attractive. Therefore, promoting clearance of toxic a-syn through active or passive immunization has also been extensively studied. A-syn active immunization was shown to result in a high-titer anti-a-syn antibody response and fewer pathologic aggregates in the striatum in animals that were vaccinated compared with animals that had received a mock vaccine [137]. Active immunization with recombinant a-syn also ameliorated a-syn related synaptic pathology in a transgenic mouse model of Parkinson’s disease [101], and passive immunization with an antibody targeting the C-terminus of a-syn in the same transgenic model reduced behavioural deficits in the water maze and axonal and synaptic deficits by promoting the lysosomal clearance of a-syn [102]. Recent studies have shown that C-terminus truncation and propagation of a-syn play a role in the pathogenesis of Parkinson’s disease/DLB. mThy1-α-syn transgenic mice, which develop symptoms resembling the motor deficits of Parkinson’s disease, were immunised with new monoclonal antibodies directed against the C-terminus of a-syn. This resulted in reduced levels of C-terminus-truncated a-syn, reduced its propagation, and ameliorated Parkinson’s disease-like pathology and improved behavioural and motor functions [51].

While it is important to reduce the global a-syn load, antibodies that could specifically target toxic aggregates would be of high therapeutic potential. In an in vitro study, C-terminal and oligomer-selective a-syn antibodies were able to reduce global a-syn levels and the extent of a-syn dimerization/oligomerization [110]. Stereotaxic administration of antibodies that target a-syn aggregates into the brains of a-syn transgenic mice prevented neuron-to-astroglia transmission of a-syn and led to increased localization of a-syn and the antibody in microglia. Furthermore, passive immunization with a-syn antibody reduced neuronal and glial accumulation of a-syn and ameliorated neurodegeneration and behavioural deficits associated with a-syn overexpression [7]. In a different study, the antibody mAb47, which recognizes a-syn oligomers, was used to treat the oligomeric load of Thy1-hA30P transgenic mice; interestingly, mAb47-treated mice showed a reduction in soluble and membrane-associated a-syn and an improvement in their motor phenotype [88]. More recently, Spencer et al. showed that single chain antibodies against a-syn oligomers delivered by lentivirus were able to reduce pathology and correct motor behaviour in mice overexpressing a-syn under the Thy1 promoter [145]. Another promising result was revealed by a screening of a novel set of AFFitopes (vaccines that use short peptides for their antigenic component) which recognised a-syn oligomers. Immunization with AFF1 in two different transgenic mouse models of Parkinson’s disease and dementia with Lewy bodies, PDGF- and mThy1-α-syn transgenic mice resulted in high antibody titers in CSF and plasma, which crossed into the CNS and recognised a-syn aggregates. This vaccination also resulted in decreased accumulation of a-syn oligomers in axons and synapses, accompanied by reduced degeneration of TH fibres in the caudo-putamen nucleus and by improvements in motor and memory deficits in both in vivo models [99]. This research has led to development of vaccines that are now in clinical trial, which is an incredibly important step for the field.

## Conclusions

If we are to understand the contribution of a-syn oligomers to Parkinson’s disease it is of utmost importance to study oligomeric pathology and its distribution at sequential stages of the disease in the human brain. This will allow the delineation of how the presence of a-syn oligomers is associated with pathological changes and may prove to be a better correlate of disease progression and severity compared to Lewy body counts.

Furthermore, as a-syn oligomers are being pursued as a blood plasma and CSF biomarker for the presence of Parkinson’s and its progression [39, 43, 152], there is a need to understand the relationship between the species found in biological fluids and those present in the diseased brain. Histopathological detection of a-syn oligomers is a key step towards a full understanding of whether the complement of a-syn oligomers in biological fluids is representative of those causing neurodegeneration in the brain.

The precise understanding of the formation, seeding and spread of a-syn oligomers in vivo is one of the key questions that remain in the field. Little by little, more light is thrown on this elusive aspect of Parkinson’s disease which is only now being interrogated. Different proteopathies often coincide in the diseased brain; in fact, it was recently shown that synthetic a-syn fibrils are able to induce tauopathy, possibly by a cross-seeding mechanism [57], and so, research regarding a-syn oligomers might not only help advance the field of Parkinson’s disease, but also that of other neurodegenerative diseases.
